# Evaluation of quantum contouring algorithms for treatment planning on MR abdominal images

**DOI:** 10.3389/fonc.2025.1553539

**Published:** 2025-08-06

**Authors:** Rachel Glenn, Tucker Netherton, Adrian Celaya, Mohamed Eltaher, Beatrice Riviere, Eugene J. Koay, David Fuentes

**Affiliations:** ^1^ Department of Imaging Physics-Research, The University of Texas MD Anderson Cancer Center UTHealth Graduate School of Biomedical Sciences, Houston, TX, United States; ^2^ Department of Imaging Physics-Research, Division of Diagnostic Imaging, University of Texas MD Anderson Cancer Center, Houston, TX, United States; ^3^ Department of Computational and Applied Mathematics and Operations Research, Rice University, Houston, TX, United States; ^4^ Department of Gastrointestinal Radiation Oncology, University of Texas MD Anderson Cancer Center Houston, Houston, TX, United States; ^5^ Sheikh Admed Center for Pancreatic Cancer Research, University of Texas MD Anderson Cancer Center Houston, Houston, TX, United States

**Keywords:** quantum computing, medical image segmentation, auto-contouring, quantum image representation, radiotherapy planning

## Abstract

**Introduction:**

Quantum computing is increasingly being investigated for integration into medical radiology and healthcare applications worldwide. Given its potential to enhance clinical care and medical research, there is growing interest in evaluating its practical applications in clinical workflows.

**Methods:**

We developed an evaluation of quantum computing-based auto-contouring methods to introduce medical physicists to this emerging technology. We implemented existing quantum algorithms as prototypes tailored for specific quantum hardware, focusing on their application to auto-contouring in medical imaging. The evaluation was performed using a medical resonance imaging (MRI) abdominal dataset, comprising 102 patient scans.

**Results:**

The quantum algorithms were applied to the dataset and assessed for their potential in auto-contouring tasks. One of the quantum-based auto contouring methods demonstrated conceptual feasibility, practical performance is still limited by current available quantum hardware and scalability constraints.

**Discussion:**

Our findings suggest that while quantum computing for auto-contouring shows promise, it remains in its early stages. At present, artificial intelligence-based algorithms continue to be the preferred choice for auto-contouring in treatment planning due to their greater efficiency and accuracy. As quantum hardware and algorithms mature, their integration into clinical workflows may become more viable.

## 1 Introduction

Research on the application of quantum computing in magnetic resonance imaging (MRI) diagnostic analysis is rapidly progressing. Microsoft’s quantum computing team has developed a quantum-inspired algorithm for magnetic resonance fingerprinting that runs on classical machines. This algorithm reduced scan times by a factor of three while increasing precision by 30% (1).

MRI works by polarizing proton spins in hydrogen atoms and measuring their relaxation times, which vary depending on the material (e.g., hard and soft tissues, bone, and cancerous cells) (2). As a quantum-based technique, MRI holds significant potential for medical imaging. Current research efforts, such as those from the Superconducting Quantum Materials and Systems Center at Fermi National Accelerator Laboratory and New York University Langone Health, aim to develop quantum algorithms for processing 3D MRI images (3). These advancements are expected to improve diagnostic speed and facilitate more accurate molecular-level analyses, which could revolutionize cancer diagnosis and treatment. Additionally, collaborations like those between the German Cancer Research Center and Fraunhofer Competence Network Quantum Computing are exploring quantum computing for personalized cancer therapies (4).

Although quantum computing promises to revolutionize medical imaging, it remains an emerging field, and its immediate impact on medical algorithms is still uncertain. In this study, we evaluated several quantum algorithms applicable to auto-contouring methods used in treatment planning systems. Our analysis found that current quantum methods for medical image processing are still in early stages of development.

A prominent contributor to the field, Stephen Jordan at Microsoft Quantum, maintains a list of quantum algorithms in his “quantum algorithm zoo” (5) Notably, research on quantum image processing (QIP) is still limited, with image segmentation representing only 3% of the quantum computing work published in this area (6, 7). Much of the literature on quantum computing has focused on security and foundational aspects of the field. However, quantum algorithms for practical applications in medical imaging are gaining traction.

Among the key quantum algorithms are Grover’s search algorithm (amplitude amplification) (8), Shor’s factoring algorithm (9), and the Harrow-Hassidim-Lloyd (HHL) algorithm (10). These algorithms are being adapted for applications such as quantum neural networks and quantum image processing. While the concept of quantum-based neural networks is still in its infancy, current research is exploring the potential of integrating quantum properties into neural network models for tasks like image contouring.

Before developing effective quantum algorithms, it is crucial to understand the limitations of the underlying quantum hardware. Different quantum devices may offer varied capabilities, and understanding these constraints will guide the development of algorithms suited for medical applications. In this paper, we evaluate three distinct quantum algorithms—quantum-inspired, gate-based, and quantum annealing-based—using an MRI dataset. We conclude with a discussion on their relative potential for auto-contouring in medical imaging.

This paper is organized as follows: We begin with a brief review of quantum vs. classical computing to provide a clearer understanding of quantum computing. The following section describes our methods for selecting the three quantum algorithms evaluated in this study. Next, we outline the dataset used for algorithm evaluation. The subsequent sections focus on three different quantum algorithms. Finally, we discuss the strengths and limitations of these algorithms, followed by concluding remarks.

## 2 Classical versus quantum computing

Classical hardware for computing is effective because of the software that runs on top of it. There are two main types of software that operate on classical hardware: operating systems and application programs. Compilers translate high-level code into bytecode and machine language, which manipulate bits (0 or 1) in the computer processor. Over the years, considerable effort has gone into developing higher-level languages that allow users to write code quickly for their applications. Similarly, software for quantum computing hardware is actively being developed. Currently, developing algorithms for quantum computers can be time-consuming because developers must tailor their algorithms to run on individual quantum bits (qubits), rather than using high-level programming languages like Rust or Python.

The rules for manipulating classical bits are very different from those for quantum bits. Five important differences between classical and quantum computations are: (1) quantum superposition, (2) interference between qubits, (3) quantum entanglement, (4) non-deterministic calculations, and (5) non-clone-ability of a qubit state.

A classical bit is either in state 0 or state 1, whereas a qubit can exist in both states simultaneously, a property known as superposition. In quantum mechanics, the state of a system is unknown until it is measured. This creates ambiguity in the use of the term “superposition.” For example, some developers describe the Hadamard operation as transforming a qubit state from $|\psi_{Q}\rangle \;=\;0|0\rangle \;+\;|1\rangle \;$ to $|\psi_{Q}\rangle \frac{1}{\sqrt{2}}=(|0\rangle +\left|1\rangle \right)$ as putting the state in a superposition of states. Note, we have employed Dirac notation representing the ket |ψ⟩ of the wavefunction. ⟨ψ|  represents the bra of the wavefunction. In quantum mechanics, there is a finite probability that a qubit can exist in superposition, even if that probability is close to zero. It is safer to assume that quantum states are always in a superposition of states until they are measured. Schrödinger’s famous cat thought experiment exemplifies this concept, where a cat inside a box is simultaneously in two states—alive and dead—until the box is opened.

Quantum entanglement is another key concept, observed in particles such as photons and electrons. It describes a scenario in which multiple particles share a wavefunction, meaning that the state of one particle directly affects the state of the other (11). This phenomenon is common in real quantum systems, qubits are not isolated and interact with their environment, other qubits, or other quantum systems. Entanglement occurs when two qubits interact and become linked in such a way that their individual states cannot be described independently. The strength of the entanglement depends on how strongly the qubits interact with each other. Weakly entangled qubits exhibit weak entanglement, while strongly entangled qubits display stronger correlations. In quantum computing, writing the entanglement between two states as ⊗  is conventional. This notation does not provide the strength of the entanglement between the two states.


$$|\psi_{a}\rangle ⊗|\psi_{b}\rangle$$

Quantum interference in quantum systems occurs when the wavefunctions of nearby qubits overlap. This commonly occurs and is one of the most challenging aspects of developing a quantum computer. When the wavefunction extends outside the physical qubit, the electron can tunnel outside the barrier containing the electron, called leakage current. When two qubit wavefunctions overlap, interference patterns emerge, similar to the ripples caused by multiple pebbles dropped in a pond. This interference can be constructive or destructive, affecting the probabilities of certain outcomes. Furthermore, when qubits are entangled, interference between their states can create a multi-level system, where the combined quantum states result in multiple possible configurations. For instance, two qubits, each with two possible states (|0⟩ and |1⟩), the combination will have four different = states: the excitation of one of the qubits is considered one state, (|01⟩ or |10⟩), while the excitation of both qubits is considered another state, (|11⟩) and both qubits in the ground state is a state (|00⟩). Mathematically, the combination of quantum states in Hilbert space forms a vector space composed of a linear combination of two or more allowed states. Control of the population of these states is the fundamental building block of quantum computer.

Quantum indeterminacy refers to the inherent uncertainty in quantum systems. Repeating the same quantum algorithm is likely to yield different results each time due to the probabilistic nature of quantum mechanics. When measuring the state of a system, the measurement itself has a probability distribution over all possible outcomes. Both the state and the measurement outcome are inherently uncertain, and this indeterminacy is a defining feature of quantum mechanics.

Cloning a quantum state—creating an exact, independent copy of a state without altering it—is not possible, due to the unique properties of quantum measurements. The act of measuring a quantum state interacts with and changes that state. Qubits in a pure state (uncoupled from the environment) can be cloned. Thus, it is impossible to measure a quantum state and then recreate the original state, unless the state were a pure state to begin with. It is worth mentioning that, although quantum cloning is impossible, it is possible to clone a state with non-perfect fidelity (12).

**Table 1** summarizes the key differences between classical and quantum computation. For further study of the basics of quantum computing, numerous online resources, books, and courses are available, including “Quantum Computation and Quantum Information” by Nielsen and Chuang (13). Further explanation of quantum computing is beyond the scope of this article, as there is a vast amount of resources available.

**Table 1:** Comparison between classical and quantum computations.

Type of comparison	Classical	Quantum
Basic Unit	Bits are either 0 or 1	Qubits are ina superposition of states
Basic Unit	Bits have a definite value	Qubits do not have a definite value until a measurement is done
Bit Manipulation	Bits are copied or read without affecting other bits’ values	Qubits are in an unknown state and cannot be copied or read without disrupting the other states. If two qubits are entangled, performing a measurement of one will affect the other qubit states
Storage	n-bit storage can hold one value from 0 to 2*^n^* − 1	n-qubits hold 2*^n^* values
Computation	n-bit processor can do one operation	n-qubit processor can do 2*^n^* operations
Computation	Operations are done via logical operators	Operations are done via Unitary Matrices
Computation	copyingabitstateis possible	Cloning a quantum or classical bit is not possible
Measurement	Calculations are deterministic; repeating the same algorithm gives the same result	Calculations are nondeterministic; for one input there can be multiple possible results

## 3 Methods

We conducted a literature search to identify all available open-source quantum contouring algorithms from GitHub and PaperswithCode, specifically focusing on algorithms for quantum image segmentation. Our search covered three different types of quantum algorithms: quantum-inspired, quantum annealing, and gate-based qubit algorithms. For each of these categories, we found one relevant algorithm. To provide concrete examples of how these algorithms can be applied, we used them for contouring the liver in MR abdominal images. This approach offers medical physicists valuable insights and a deeper understanding of how quantum algorithms can be utilized within treatment planning systems.

In this study, all quantum algorithms were implemented and evaluated using quantum simulators rather than physical quantum hardware. Simulators allow for idealized performance assessment of these methods for possible use as quantum image segmentation methods. While this provides a useful framework of the initial algorithm development, it doesn’t capture the challenges with deploying these on quantum hardware. Future work will need to incorporate experimental validation on physical quantum devices to better understand the true capabilities and limitations of quantum auto-contouring in clinical applications.

## 4 Dataset

The dataset consisting of 34-patients, with a total of 102 scans, consisting of MR imaging data of the abdomen, with unenhanced, late arterial phase, and portal venous phase. Imaging was acquired from multiple MR scanners: Optima MR450w,1.5T, (GE), TrioTim, 3T, (Siemens), Intera, 1.5T (Philips), Achieva 1.5T (Philips), and Panorama 1T (Philips). Preoperative abdominal MR was obtained using a liver protocol consisting of an arterial phase (20–30 seconds after contrast injection), pre-contrast phase, a venous phase (60–80 seconds after injection of intravenous contrast material), and a delayed phase (15 minutes after contrast injection). Each series was obtained in the axial plane with a phased array multi-coil. A T1-weighted spoiled gradient echo sequence was performed. The multi-phase MR studies were exported in DICOM format from the picture archiving and communication system to an independent server running 3D Slicer (14). Data collection and analysis for this study were conducted in accordance with Institutional Review Board (IRB) protocols PA15-0091, titled Image Processing for Retrospective Analysis of Therapy Response and Planning, under the approval of Ada Lo at MD Anderson Cancer Center, Houston, TX. All experiments were carried out in accordance with institutional policies. PHI was removed. The imaging data was analyzed as NifTI files. The liver was contoured by a radiologist in 3D Slicer. All images were pre-processed using SimpleITK (15) for non-uniformity in the main magnetic field, (16). The 3D Slicer scripting interface was used to iteratively anonymize all data and maintain orientation and resolution information as a compressed NifTI format recommended by the Neuroimaging Informatics Technology Initiative.

This dataset contains demographic and clinical information primarily focused on liver-related conditions such as hepatocellular carcinoma and cirrhosis. The patient cohort has a mean age of approximately 66 years, with ages ranging from 44 to 92. Most patients are male (82%), with females representing 18% of the sample. Racial composition is diverse, including individuals identified as White (52%), Asian (21%), Black (9%), Hispanic (6%), and unknown (12%). This dataset offers a rich foundation for analyzing patterns in liver disease across a varied patient population, with cleanly structured demographics that support stratified analysis by age, gender, and race.

## 5 Evaluation of quantum inspired classical algorithm

QIS-Net (Quantum Image Segmentation Network) is a self-supervised, quantum-inspired machine learning algorithm designed for image segmentation (17). It uses an image threshold parameter for each pixel, defining a quantum-like state based on an individual pixel and its eight nearest neighbors through fuzzy logic. In this framework, each pixel neighborhood is mapped into a three-level quantum-like system (qutrit), where the levels represent low, medium, and high intensities derived from fuzzy membership functions.

The self-supervised approach relies on unlabeled data, using learned representations for feature detection and classification from raw input. Instances of self-supervised approaches include: GPS location data to distinguish between objects typically found in buildings, schools, homes, etc.; hashtags in online content to assist machine learning in environments with limited labeled data; and techniques like edge detection and super-pixels in images to help train neural networks using unlabeled data.

**Figure 1:**
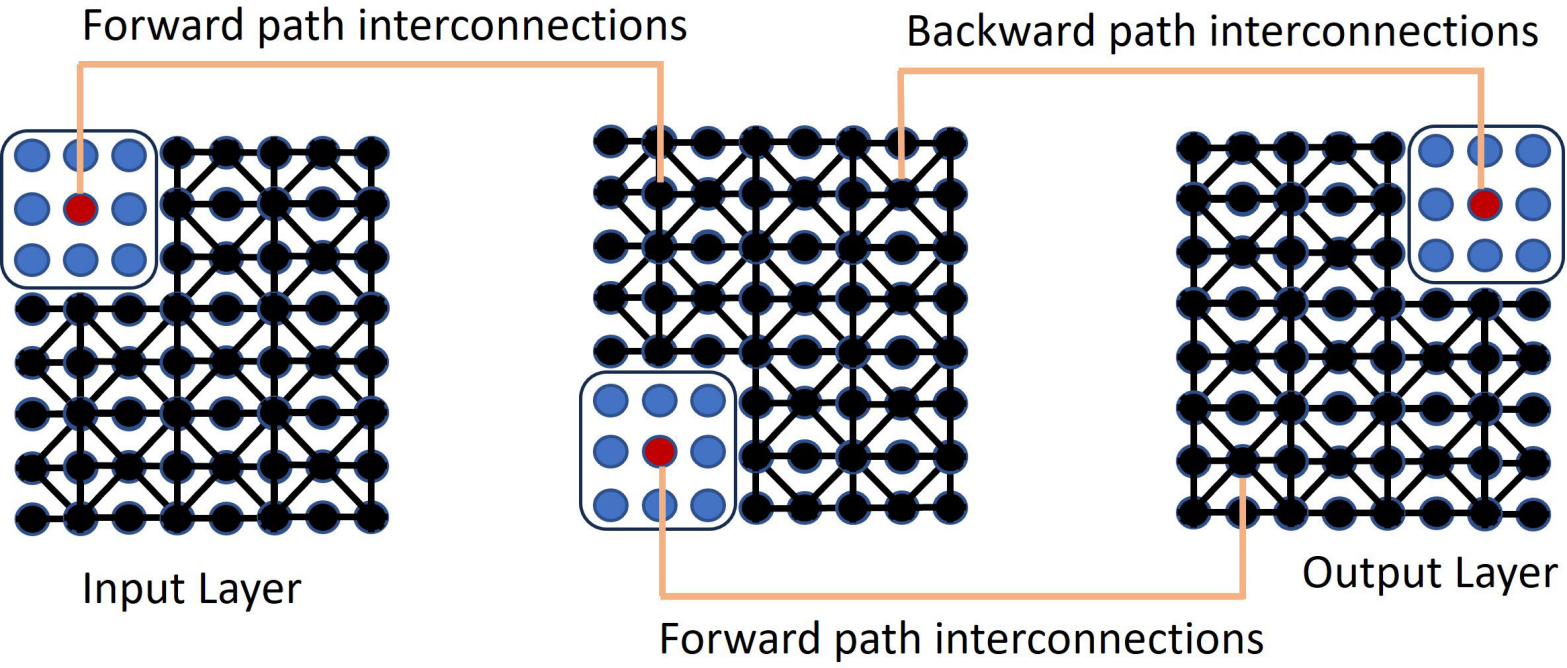
The self-supervised QIS-Net neural network.

The biggest challenge with fully unsupervised learning is making sure that the derived data representation and the learning tasks are aligned. In QIS-Net, rotational gates are applied to simulate unitary evolution of each qutrit. The first convolutional layer is constructed by applying a counter-clockwise rotation operation R(θ) to each qutrit set, and the second layer by applying a clockwise rotation operation R(-θ). These rotations are modeled as unitary transformations in the qutrit Hilbert space, approximating superposition interactions among neighboring pixel intensities. QIS-Net is shown in **Figure 1**.

The completion of the image segmentation algorithm was done by measuring the population |⟨*z*|*z*⟩|^2^, where *z* represents the simulated quantum state associated with a pixel neighborhood. The input-output relation of the *j*th quantum pixel at the center of the qutrit to the *t*th quantum pixel at the center of the qutrit on the same image was defined through the calculated transition probabilities derived from these population measurements


$${\mathrm{|z}}_{t}^{k}(x_{t})\rangle =\text{σ}\left(x_{t}\right)\sum_{j}^{n\times \text{m}}e^{iz_{j}^{k-1}}\langle \phi_{j}^{t}{\text{|η}}_{j}^{t}\rangle ,$$

where *z_t_* is the output quantum neuron. In essence, |⟨*z*|*z*⟩|^2^, converts the quantum information, which is on a classical machine, into classical information by collapsing the wavefunction. The parameter *x_i,j_* is the *i*th neuron in the image to be sampled


$$x_{t,j}=\sum_{p-1}^{m-2}\sum_{c-1}^{1}\mu_{+p,+c}\cos\left(\omega_{t,j}-A_{t,j}\right)$$

where $A_{i,j}=2\pi B_{i,j}$ , and $B_{i,j}\;$ is the cardinality of the image, which is the sum of the intensities of the pixels that compose the *t*th qubit. The summation, *j*, is over the eight surrounding pixels. To determine whether it was foreground or background, a quantum sigmoidal activation function was applied, given as


(1) $$\text{σ}\left(x\right)=\frac{1}{{\text{λ}}_{\text{ω}}+e^{-\text{ν}\left(x-\text{β}\right)}},$$

where *ν* is the steepness factor, *β* the activation factor, and *λ_ω_* the response of a gray scale intensity index. The frequency is given by


$${\text{ω}}_{i,j}=2\text{π}\left(2\text{π}-\sum_{p=-1}^{m-2}\sum_{c-1}^{1}{\text{μ}}_{i+p,j+c}-\mu_{i,j}\right);\;j\in \left\{1,2,\ldots 8\right\}$$

The gray scale intensity index is given as


$${\text{λ}}_{\text{ω}}={\text{Σ}}_{\text{ω}}\frac{S_{N}}{F_{{\text{λ}}_{\text{ω}}}-F_{{\text{λ}}_{\text{ω}-1}}},$$

where *S_N_* is the sum of the 8-connected neighboring grayscale pixels values as qubits. The interconnection strengths, *ϕ_ij_* between two adjacent layers were inspired by a quantum fuzzy membership between neurons *i* and *i*′ in different layers as


$${\mathrm{|\phi}}_{k,j}^{t}\rangle =\left[\begin{array}{c} \cos\left(\alpha_{k,j}\right)\\ \sin\left(\alpha_{k,j}\right) \end{array}\right];\;j\in \left\{1,2,\ldots 8\right\}.$$

The index *j* is over the 8 surrounding pixels that make up the qutrit. The relative measure of quantum fuzzy membership enables the network to detect the edges between foreground and background pixels


$$\alpha_{kj}=\frac{\text{π}}{2}-\left({\text{μ}}_{k}-{\text{μ}}_{kj}\right);j\in \left\{1,2,3,\ldots ,8\right\},$$

where *µ_k_* is the pixel intensity of the *k*th neuron and *µ_k,j_* one of its’ eight-neighbors *j* ∈ {1,2*,…*8}. The threshold parameter *η_k_^t^* is given as ${\mathrm{|\eta}}_{k}^{t}=\left[\begin{array}{c} \cos\left(\gamma_{k}^{j}\right)\\ \sin\left(\gamma_{k}^{j}\right) \end{array}\right]$ where ${\text{γ}}_{k}^{t}=2\text{π}\sum_{j}{\text{μ}}_{t,j}$ . The translation of an individual pixel and its’ eight-nearest neighbors into a qubit was done using a multi-class gray-level transition for a fixed class $F^{\lambda_{\omega L}}\in \left[0,\frac{\pi }{2}\right]$ . The multi-class gray-level transitions considered were


$$\begin{array}{c} F_{{\text{λ}}_{\text{ω}L}}=\frac{\text{π}}{2}\{\left[\;0,\;0.15,\;0.30,\;0.46,\;0.61,\;0.76,\;0.91,\;1.0\right]\\ \left[0,0.14,0.28,0.42,0.56,0.70,0.90,1.0\right]\\ \begin{array}{c} \left[0,0.13,0.26,0.39,0.52,0.75,0.91,1.0\right]\\ \left[0,0.18,0.36,0.54,0.72,0.90,0.97,1.0\right]\\ \begin{array}{c} \left[0,0.17,0.34,0.51,0.68,0.85,0.95,1.0\right]\\ \left[0,0.15,0.29,0.43,0.57,0.71,0.85,1.0\right]\\ \begin{array}{c} \left[0,0.15,0.30,0.46,0.61,0.76,0.91,1.0\right]\\ \left[0,0.62,0.71,0.80,0.83,0.93,0.98,1.0\right]\\ \begin{array}{c} \left[0,0.60,0.72,0.82,0.94,0.96,0.98,1.0\right]\\ \left[0,0.63,0.74,0.79,0.82,0.88,0.97,1.0\right]\\ \left[\;0,\;0.70,\;0.74,\;0.79,\;0.82,\;0.88,\;0.98,\;1.0\right]\}. \end{array} \end{array} \end{array} \end{array} \end{array}$$

The user modified the parameters *ν* and *F_λωL_* (multi-threshold) for optimum results. The investigators calculated a Dice similarity coefficient (DSC) of up to 0.83. The images contained varying levels of quantization corresponding to different features in the image. A post-processing step was performed to remove unwanted nearby features. The authors evaluated the self-supervised quantum neural network on 800 MR images of brain tumors and calculated the Dice similarity score. The DSC for QIS-Net was 0.781, compared to U-Net’s score of 0.991 on the same dataset.

**Figure 2:**
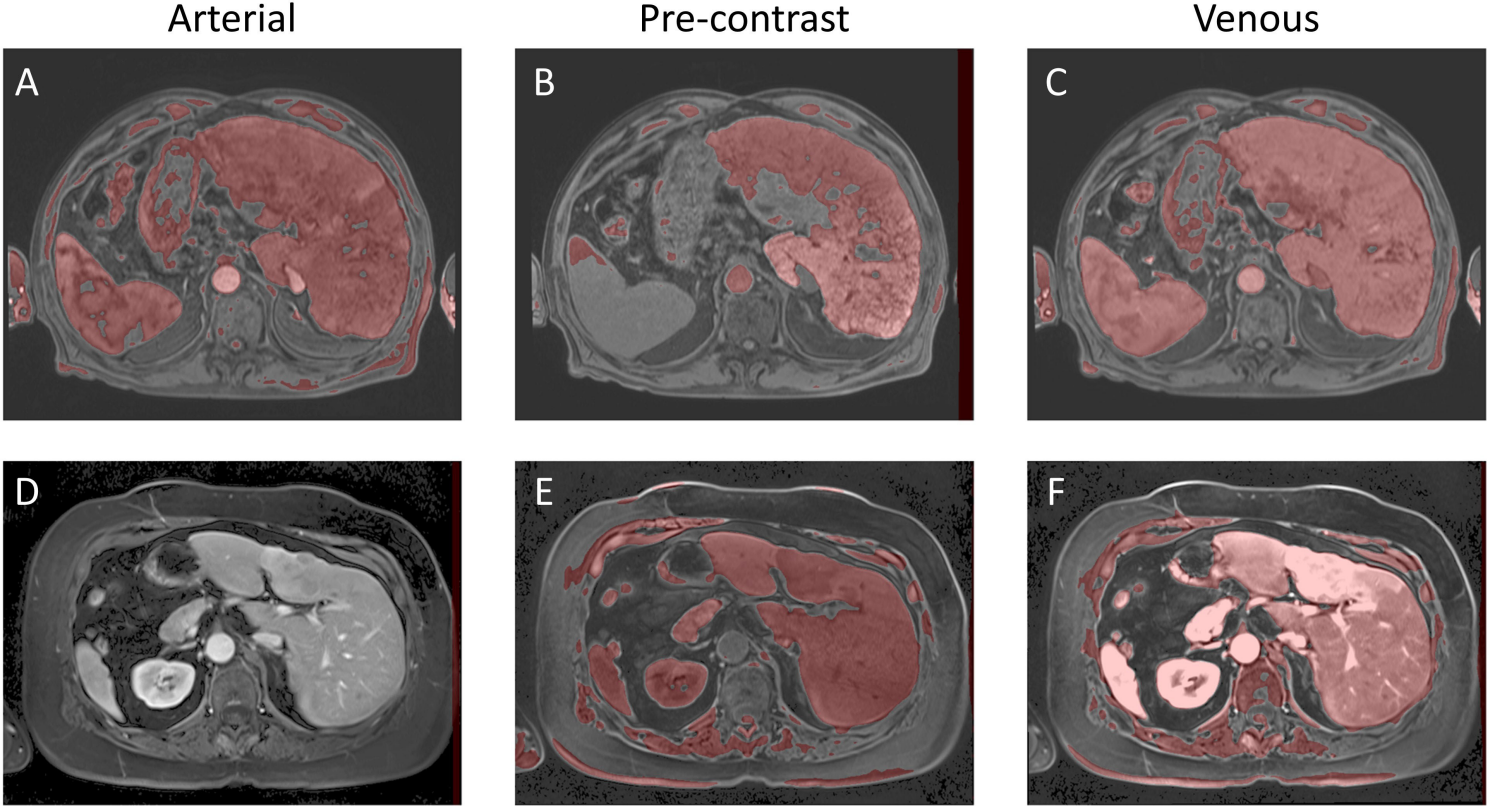
Example image segmentation of the liver using QIS-Net. Top row **(A–C)** Patient 21 (Arterial), Patient 22 (Pre-contrast), and Patient 23 (Venous). Bottom row **(D–F)** Patient 1 (Arterial), Patient 2 (Pre-contrast), and Patient 3 (Venous).

**Figure 3:**
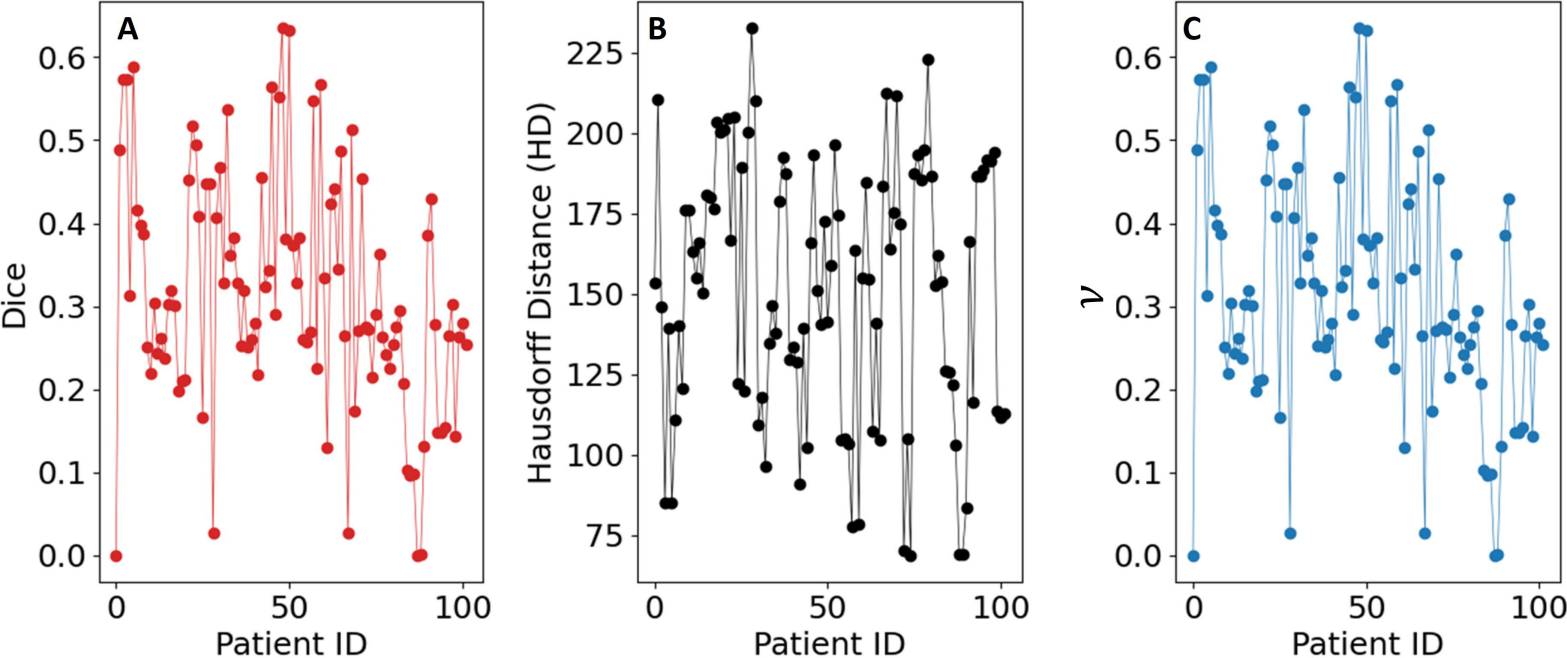
Evaluation metrics for the QIS-Net algorithm on the dataset: **(A)** Sørensen–Dice coefficient, **(B)** Hausdorff distance, and **(C)** optimized values of ν.

The available code was written to vary the steepness parameter (*ν*) in Equation 1 and allowed the user to select the optimum image from an array of images. QIS-Net claimed to use a self-supervised learning framework in which the segmentation predictions were done without the need for pixel-level supervision during each training iteration. While this approach worked for the authors, we found that we needed to optimize the algorithm over a set of parameters. We modified the code to calculate the Sørensen-Dice coefficient,


(2) $$DSC=\frac{2\left|X\cap Y\right|}{\left|X\right|+\left|Y\right|},$$

for each value of *ν* ∈ [0.02, 0.03, 0.04, 0.05] that was utilized for each patient. As part of the postprocessing step, we extracted the largest connected component and applied hole filling to the predicted binary segmentation from the predicted binary image. The value of *F_λωL_* that gave the highest Dice coefficient was consistently [0, 0.04, 0.28, 0.42, 0.56, 0.70, 0.90, 1.0]. As a final post-processing step, we added an Otsu thresholding of 4 levels and selected the highest threshold value. Example image segmentation for two patients over all three phases are shown in **Figure 2**. The resulting Sørensen–Dice coefficient (a), Hausdorff distance (b), and optimized values of *ν* (c) are presented in **Figure 3**. The median Dice score was 0.3144 ± 0.1428 and Hausdorff distance (HD) coefficient 151.1784 ± 40.7488. The code was written in MATLAB R2021b and run on an NVIDIA GPU Quadro RTX 6000. The cloned and modified source code is available on GitHub: https://github.com/rachelglenn/QISNET.

## 6 Evaluation of quantum image segmentation on a gate-based quantum computer

**Figure 4:**
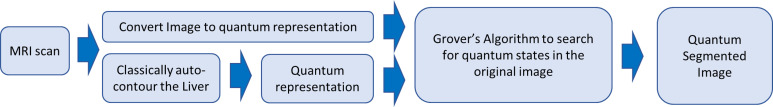
Diagram of Grover-based image segmentation.

Grover’s search algorithm provides a method to efficiently identify marked elements within a large, unstructured dataset. In this study, Grover’s method was adapted to the task of medical image segmentation following the framework presented in Ref. (18) by treating the image as a quantum state and searching for pixels belonging to the target organ structure. An overview of this Grover-based segmentation approach is illustrated in **Figure 4**.

A lightweight U-Net model was first used to generate a preliminary segmentation, identifying candidate pixels likely to represent liver tissue. These candidates were designated as marked states within the quantum system.

Three-dimensional medical image segmentation requires significant memory resources for classical storage. A 32-bit image of size 1024 x 1024 x 72 requires approximately 2.42 Gigabits (around 300 Megabytes) of storage. In contrast, quantum image representation (QIR) techniques such as the Normal Arbitrary Quantum Superposition State (NAQSS) (19), offer a highly compact alternative. It can represent the same multi-dimensional image using only (*n*+1)-qubits, where *n* encodes pixel location, and the additional qubit encodes intensity. For an image of this size, NAQSS would require approximately 28 qubits, offering a highly compact representation that could significantly reduce memory requirements if large-scale fault-tolerant quantum computers become available.

In this study, the medical image and the AI-marked candidates were encoded into a quantum state. Super-pixel intensities were normalized, and the spatial coordinates were mapped onto the NAQSS representation. A Hardamard gate was used to place the quantum image in a superposition of states. Grover’s search algorithm then operates by repeatedly rotating the quantum state’s probability amplitudes to amplify the likelihood of measuring marked candidates. An initial equal superposition of all pixel states is prepared using Hadamard gates. Further, Grover is composed of an oracle function and diffusion operator. The oracle inverts the phase of the marked states by using a Z-quantum gate. The diffusion operator is composed of three gates the Hadamard, Pauli-X, and Controlled-Z gates. It reflects the entire state about the mean amplitude. Repeating both operators increases the probability of the marked states.

We walk the reader through the encoding process that maps a classical image into a quantum representation using the NAQSS (Normalized Amplitude Quantum Superposition State) model, which is a precursor step to applying Grover’s search algorithm for segmentation. A detailed derivation of Grover’s algorithm can be found in (13); here we focus on the adaptation of Grover’s search to medical image segmentation. All operations performed classically in MATLAB without modeling physical noise or hardware constraints.

In NAQSS, the image is mapped into a superposition of quantum states where each basis state |i〉 encodes the position of the pixel and $\theta_{i}\;$ encodes the pixel intensity. For an image with 2*^n^* pixels, the image will require *n* + 1 qubits (18).


$$|\psi_{2}\rangle =\theta_{i}|i\rangle$$

The spatial image coordinates are expanded by horizontal and vertical locations (*x* and *y*) using $|i\rangle =|x_{m}\rangle |y_{k}\rangle$ .


$$|i\rangle =|x_{m}\rangle |y_{k}\rangle =|i_{n}.i_{k+1}\rangle |i_{k}\ldots i_{1}\rangle$$

where the *x*-axis and *y*-axis of the image are represented as $\left|x_{m}\rangle \;=\right|i_{n}\;\ldots \;i_{k+1}\rangle$ and $\left|y_{m}\rangle \;=\right|i_{n}\;\ldots \;i_{k+1}\rangle$ .

The intensity is normalized to define the amplitude as


$$\theta_{i}=\frac{a_{i}}{\sqrt{\sum_{y=0}^{2^{n}-1}a_{y}^{2}}},$$

where *a_i_* defines the color


$$a_{i}=\frac{\pi }{2}\left(\frac{i-1}{M-1}\right)$$

with *i* ∈ {1,2*,…,M*}. *M* represents the total number of intensity levels. This step embeds the entire image into a quantum superposition, similar to how classical images are store in RAM but compressed into a quantum state.

Grovers algorithm requires that each pixel be labeled as foreground or background using a segmentation mask—either predefined or AI-generated.

For segmentation, the image is divided into foreground and background components. This is done by attaching a label qubit $|{\text{χ}}_{i}\rangle$ to each state:


$$|{\text{χ}}_{i}\rangle =\{\begin{array}{c} |0\rangle ,\;\text{background}\\ |1\rangle ,\;\mathrm{foreground}\; \end{array}$$

The full quantum image encoding for Grover’s algorithm can be written as


(3) $$|\phi_{\chi }\rangle =\theta_{i}|i\rangle |{\text{χ}}_{i}\rangle =\sum_{i\in B}{\text{θ}}_{i}|i\rangle |0\rangle +\sum_{i\in F}{\text{θ}}_{i}|i\rangle |1\rangle ,$$

where *B* is the background and *F* is the foreground. The binary label helps Grover’s algorithm focus its search of pixels in the image on “marked” foreground states.

Rather than checking for a state individually, as with a classical algorithm, Grover’s algorithm searches across the entire image in a superposition of all possibilities, iteratively, amplifying pixels labeled as foreground and destructively interferes the background states. The measurement after the optimal number of iterations of Grover’s algorithm is given by


$$|\psi_{g}\rangle =\frac{\sum_{i\;\in A}a_{i}|i\rangle |1\rangle }{\sqrt{{\text{Σ}}_{i\in A}a_{i}^{2}}}$$

The foreground image is obtained with the probability


$$\text{Pr}\left(\psi_{g}\right)=\sum_{i\in A}a_{i}^{2}.$$

The only remaining requirement to run Grover’s search algorithm is identifying the marked (foreground pixels) quantum states. We use an AI-based segmentation mask for the marked states. This framework demonstrates how a quantum—enhanced search can be applied to medical image segmentation for detecting labeled structures, such as the liver. NAQSS provides a compact and physically meaningful way to encode spatial and intensity information into a quantum state to enable quantum algorithms, such as Grover’s search algorithm.

### 6.1 Method to mark quantum states

We trained a U-Net PocketNet architecture (20) neural network to generate the marked quantum state of the liver in abdominal images. The MR images were pre-processed using z-score intensity normalization. A 5-fold cross-validation training method, where in each fold 80% of the data for training and 20% for testing, ensuring no patient overlap between folds.

In the K-fold method, the dataset is shuffled and split into K groups. One group is held out as the testing dataset, while the remaining K−1 groups are used for training. The model is evaluated on the held-out testing group, and this process is repeated such that each group serves as the testing dataset exactly once.

Within each fold, the training dataset was further split into training and validation subsets (80/20 split). Training utilized the Adam optimizer with an initial learning rate of 0.001 (21). During training, if the loss plateaued for 10 steps within an epoch, the learning rate was reduced by a factor of 0.9. The sliding window approach was used, with a patch size of 256×128×128. Each K-fold dataset was trained for 200 epochs with 250 steps per epoch. In the postprocessing, we extracted the largest connected component and applied hole filling to the predicted binary segmentation. At the end of each epoch, the Sørensen-Dice Coefficient (SDC), as defined in Equation 2, was calculated using the validation dataset. Training was performed using TensorFlow v2.8.0 on an NVIDIA Quadro RTX 8000. The network weights were initialized with TensorFlow’s default values.

**Figure 5:**
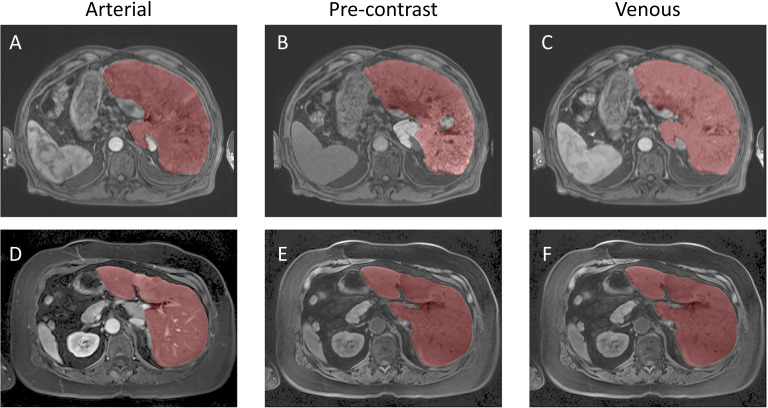
Example Image Segmentation using classical AI. Top row **(A–C)** Patient 21 (Arterial), Patient 22 (Pre-contrast), and Patient 23 (Venous). Bottom row **(D–F)** Patient 1 (Arterial), Patient 2 (Pre-contrast), and Patient 3 (Venous).

**Figure 6:**
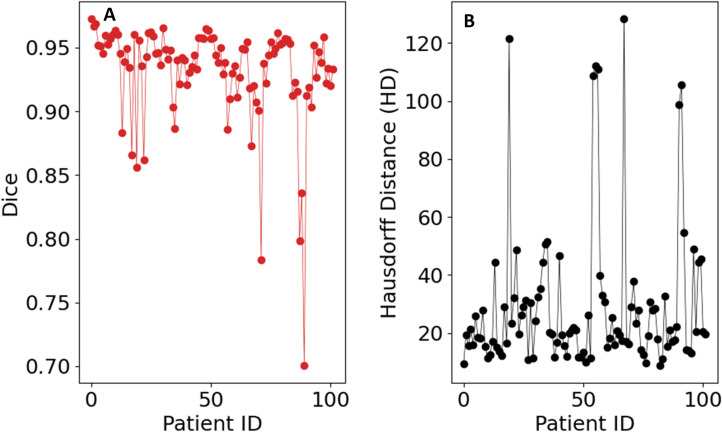
Evaluation metrics for the UNet-PocketNet algorithm on the dataset: **(A)** Sørensen–Dice coefficient, **(B)** Hausdorff distance.

Inference was conducted on the testing dataset after each K-fold training session. A sliding window approach was applied during inference, with the same patch size as used in training. The window was moved in steps equal to half the patch size, and predictions were weighted with a Gaussian kernel having a full-width half-maximum (FWHM) of 0.029 pixels. The largest connected components in each image were selected, and the Dice score was computed for each patient in the testing dataset at the end of each training cycle. Example image segmentation of two patients are shown in **Figure 5**. The Dice scores and HD coefficients are shown in **Figure 6**. The Dice median across all folds was 0.9307 ± 0.0383 and HD coefficient 29.0499 ± 25.0915. The median Dice score and standard deviation was aggregated across the testing datasets from all five folds. For each fold, Dice scores were calculated on the held-out testing subset, and the distribution of these scores across all patients was used to compute the overall median and standard deviation. This provides a summary of model performance variability across the entire dataset.

### 6.2 Results of Grover’s algorithm using the marked states

**Figure 7:**
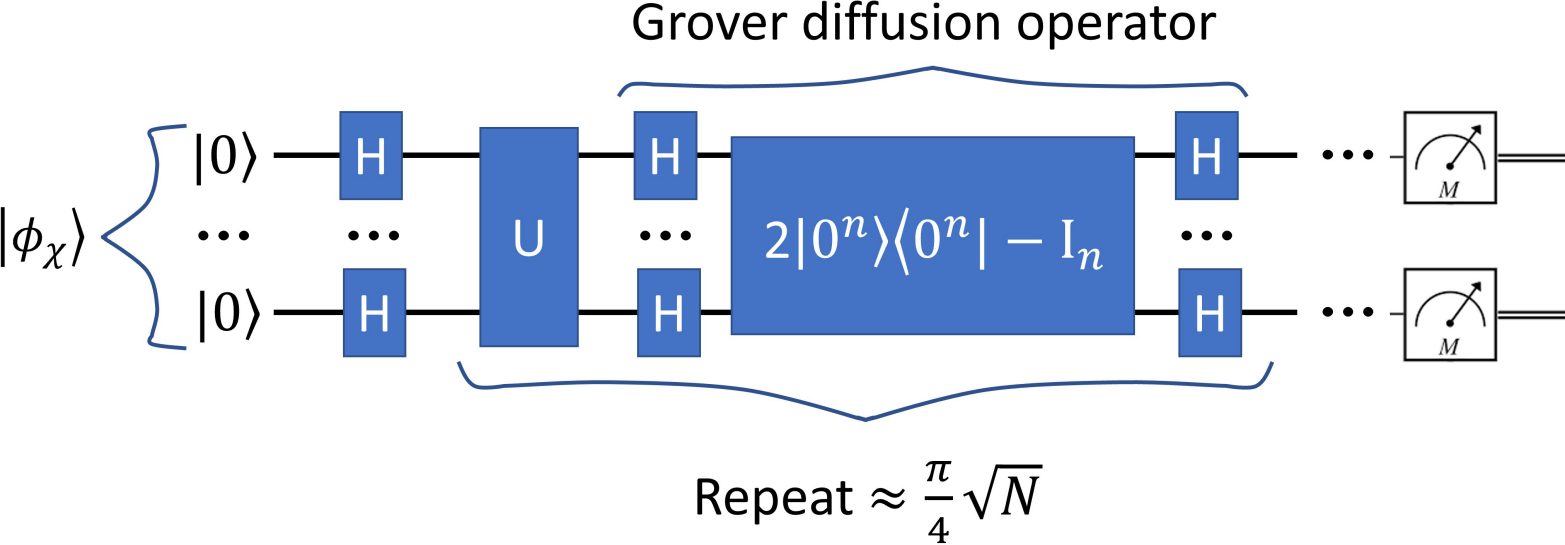
The quantum circuit representing Grover’s algorithm. The input image is represented expressed in Equation 3. The output is represented via a measurement.


Grover quantum circuit is illustrated in **Figure 7**. The trained model was used to mark states for Grover’s search algorithm, which was implemented classically in MATLAB. To reduce the computational complexity, we utilized super-pixels to limit the number of pixels being searched. The optimal number of iterations for Grover’s algorithm was determined using the formula (18):

(4) $$t\;=\;\frac{1}{\omega }\left(\;\frac{\pi }{2}\;-\;\phi \right)$$

where


$$\omega =\cos^{-1}(1-2\frac{r}{N}),$$

and


$$\phi =\tan^{-1}\left(\frac{A_{0}}{L_{0}}\sqrt{\frac{r}{\left(N-r\right)}}\right).$$

Here, *A*_0_ and *L*_0,_ represent the average amplitudes of the marked and unmarked states, respectively. The variable *r* is the number of non-zero pixels, and N is the total number of pixels in the image.

**Figure 8:**
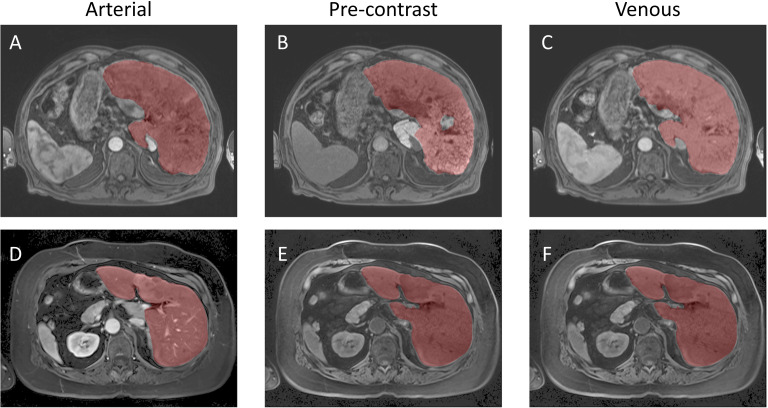
Example image segmentation using the hybrid Grover’s/AI algorithm. Top row **(A–C)** Patient 21 (Arterial), Patient 22 (Pre-contrast), and Patient 23 (Venous). Bottom row **(D–F)** Patient 1 (Arterial), Patient 2 (Pre-contrast), and Patient 3 (Venous).

**Figure 9:**
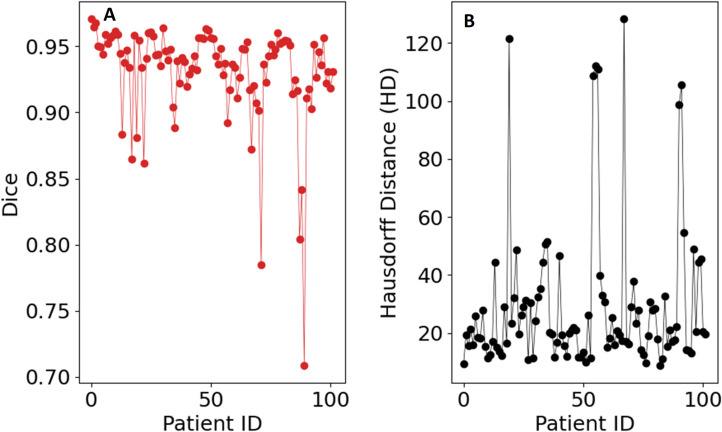
Evaluation metrics for the hybrid Grover’s algorithm on the dataset: **(A)** Sørensen–Dice coefficient, **(B)** Hausdorff distance.

The source code for the implementation is available on GitHub at https://github.com/rachelglenn/QIS. Calculations were executed on an NVIDIA GPU Quadro RTX 6000. Example image segmentations for two patients across all three phases are shown in **Figure 8**. In the postprocessing, we extracted the largest connected component and applied hole filling to the predicted binary segmentation. The Dice coefficient and HD distanced can be seen in **Figure 9**, with a median score 0.9309 ± 0.0400 and HD coefficient 29.0499 ± 25.0915. The Dice score for the QCuts algorithm was not subject to cross-validation and was calculated for the entire dataset. The optimal number of iterations was determined using Equation 4. As this was simulated without quantum noise, convergence followed the expected theoretical pattern.

## 7 Evaluation of an annealing-based quantum algorithm

QCUTS (Quantum cuts) is a quantum annealing-inspired method for image segmentation by formulating the task as an energy minimization problem (22). Quantum annealers are quantum hardware that find a global minimum to an objective function. In this approach, super-pixels are used to reduce the dimensionality. The super-pixels are modeled as nodes in a graph and the edges encode a similarity measure based on the pixel intensity differences. Optimal segmentation occurs by minimizing the total energy of the system. This process is mathematically analogous to solving the Schrödinger equation for eigenvalues.

Quantum annealing (23) leverages quantum effects such as entanglement and superposition to find the lowest-energy state of a Hamiltonian (cost function). This process has been successfully applied to problems like the traveling salesman problem, scheduling tasks, and graph-based optimization. It begins by initializing the qubits in the lowest-energy state of a simple Hamiltonian. By adjusting the Hamiltonian parameters (by using tunneling and superposition) the system evolves and explores a vast solution space and ultimately settles into the lowest-energy state of the target Hamiltonian, corresponding to the optimal solution of the problem. This method is particularly well-suited for solving graph-based image segmentation problems, where the image is mapped onto an energy equation with two states: foreground and background. Compared to gate-based quantum computers, the D-Wave quantum annealer excels in solving such problems due to its design and optimization capabilities.

In this study, QCUTS was adapted here to evaluate its utility on MR abdominal images. It was implemented using classical simulated annealing to approximate quantum annealing behavior; no real quantum hardware was employed. Future evaluations on physical quantum annealers, such as D-Wave systems, may differ due to factors like noise, limited qubit connectivity, and decoherence.

The following sections describe the energy formulation used in QCUTS and evaluate its performance on liver segmentation tasks.

### 7.1 The QCuts algorithm

**Figure 10:**
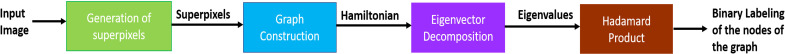
QCUITS quantum-classical image segmentation algorithm.

QCuts is a quantum-enhanced method for graph-based image segmentation, as illustrated in **Figure 10** (22). Note that graph-based image segmentation algorithms are naturally well-suited for high-contrast grayscale or black-and-white images. This is because they rely on intensity differences and edge detection to construct graphs and identify regions, tasks that are simpler when contrast is pronounced.

The algorithm begins by taking an input image and generating super-pixels, which were treated as nodes in a fully connected graph. Edges between nodes were weighted according to the grayscale intensity similarity between the corresponding super-pixels, with stronger weights assigned to more similar regions. The labeling vector is derived from the Hadamard product of the eigenvalues, reflecting quantum principles. Note that in this context, “Hadamard product” refers to the classical element-wise multiplication between vectors, and is not the quantum Hadamard gate often used to create superposition in quantum computing. This classical product formulation was inspired by quantum notation but does not involve actual quantum operations.

The energy expression consists of two terms:


(5) $$E\left(y\right)=E_{\text{unary}}\left(y\right)+\lambda E_{\text{binary}}\left(y\right).$$

A unary potential, $E_{unary}$ a binary potential $E_{binary}$ . The unary term encourages super-pixels believed to belong to the target organ or structure to be labeled accordingly. It was defined as a simple binary indicator based on initial seed selection from low-intensity regions. The parameter λ controls the weight contribution of the binary term compared to the unary term. It was assumed that *λ* = 1.

The unary term is defined as


$$E_{\text{unary}}\left(z\right)=\frac{1}{{\text{Σ}}_{i}\left(z_{i}^{2}\right)}\left\{ \begin{array}{l} 1,\text{ if }S_{i}\;\in S_{\text{foreground}}\\ 0\text{, otherwise.} \end{array}\right.,$$

where $S_{i}$ denotes the ith super-pixel and $S_{\text{foreground}}\;$ represents the set of super-pixels with the lowest mean grayscale intensity values, assumed to correspond to the target structure.

The binary potential promotes smoothness by penalizing label differences between similar neighboring super-pixels, based on a Gaussian-weighted intensity difference:

$E_{\text{binary}}\left(z_{i},\;z_{j}\right)=\;\frac{1}{{\text{Σ}}_{i}\left(z_{i}^{2}\right)}w_{i,j}\left(z_{j}^{2}-z_{i}z_{j}\right)$ measures how appropriately labeled the pixel, *z_i_*, is given the image information. To ensure minimization, the labeling vector **z** is replaced by **y=z** · **z**, which has the values [-1,0,1].

The Gaussian weight is defined as


$$w_{ij}=\frac{1}{\text{σ}\sqrt{2\text{π}}}\exp\left(-\frac{{\left|\left|S_{i}-S_{j}\right|\right|}_{2}}{2\sigma^{2}}\right),$$

where


$$S_{i}=\frac{{\text{Σ}}_{k}{\text{δ}}_{ik}I_{k}}{{\text{Σ}}_{k}{\text{δ}}_{i}k}$$

**Figure 11:**
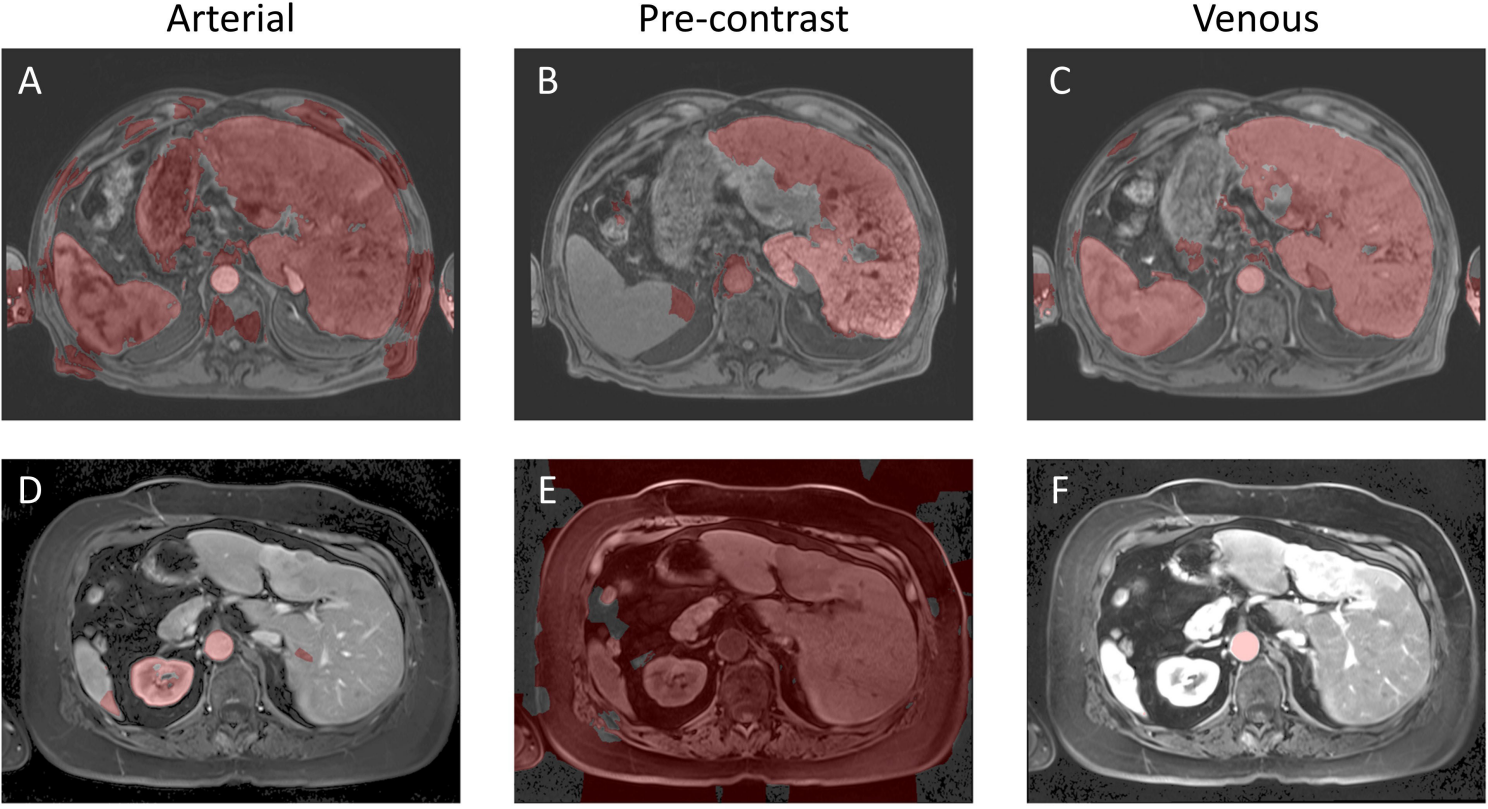
Example image segmentation using the QCuts method. Top row **(A–C)** Patient 21 (Arterial), Patient 22 (Pre-contrast), and Patient 23 (Venous). Bottom row **(D–F)** Patient 1 (Arterial), Patient 2 (Pre-contrast), and Patient 3 (Venous).

**Figure 12:**
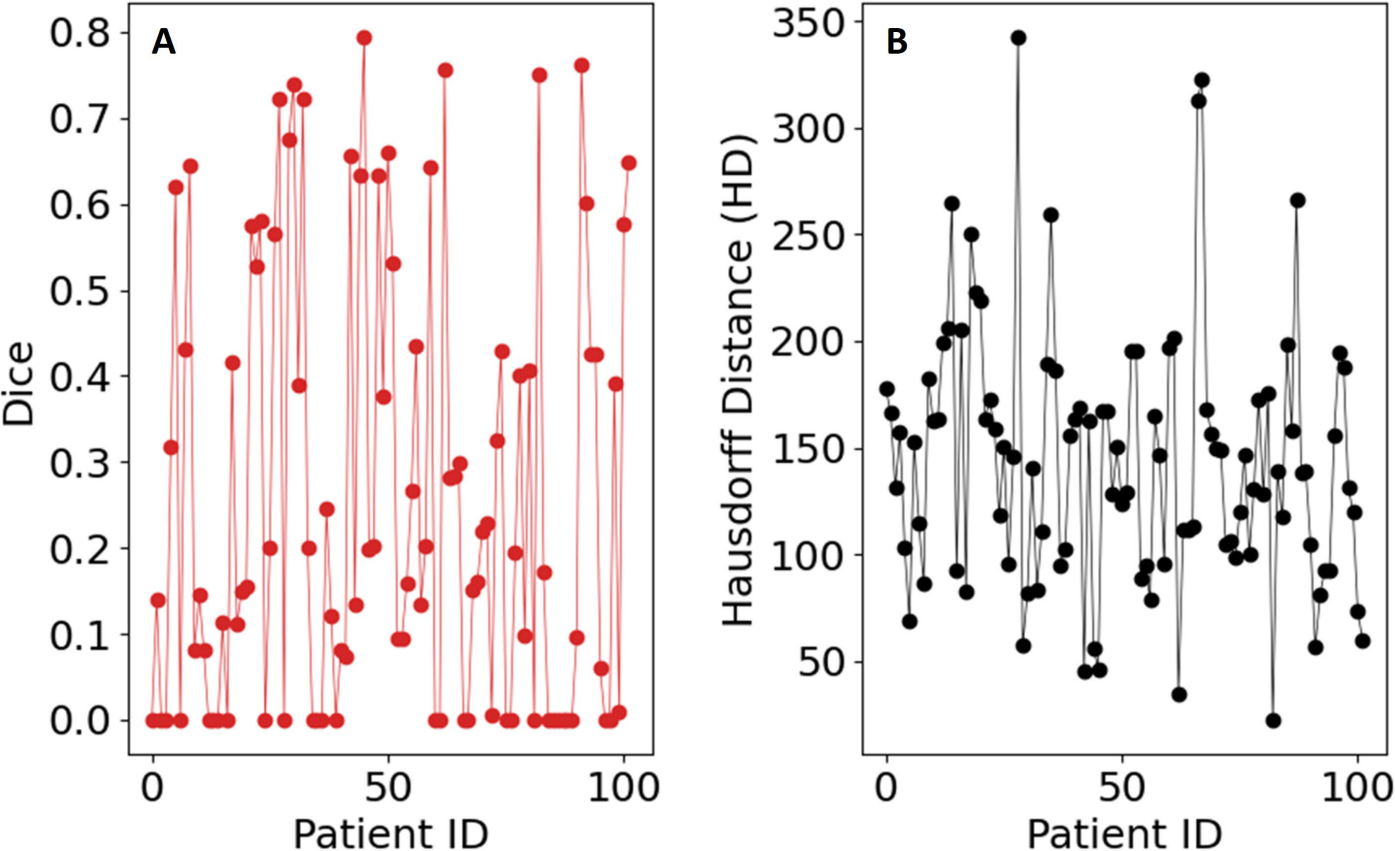
Evaluation metrics for the QCUTS algorithm on the dataset: **(A)** Sørensen–Dice coefficient, **(B)** Hausdorff distance.

The delta function *δ_ik_* indicates whether or not the *k*th voxel belongs to the *i*th super voxel. The optimal solution of Equation 5 was obtained by computing the eigenvectors corresponding to the smallest eigenvalues using MATLAB’s built-in linear algebra solvers. We then calculated the Dice coefficient, Equation 2. Example image segmentation of two patients are shown in **Figure 11**. The cloned and modified source code is available on github, https://github.com/rachelglenn/qcuts3D. Calculations were executed on an NVIDIA GPU Quadro RTX 6000. The Dice coefficient and HD distance are shown in **Figure 12**. The Dice score median was 0.2533± 0.2529 and HD coefficient 143.348± 59.6341. The low dice score can be attributed to several factors, including the algorithm’s reliance on grayscale intensity differences, which are less pronounced in abdominal MR images compared to higher contrast images such as porous media. Additionally, the lack of anatomical priors or AI guidance makes QCuts susceptible to errors in defining target structures.

Future studies on actual quantum annealers, such as D-Wave, will need to carefully assess how noise, limited qubit connectivity, and hardware-induced stochasticity affect segmentation accuracy and repeatability. For example, a QCuts implementation mapped onto a D-Wave system would require approximately 256 logical qubits if the image is preprocessed into 256 superpixels. Due to the need for minor embedding on D-Wave’s sparse qubit architecture, the actual number of physical qubits could range from 384 to over 2500, depending on graph connectivity and embedding efficiency. These hardware constraints may influence both the feasibility and stability of segmentation outcomes and must be addressed in future work.

## 8 Discussion

Developing quantum segmentation methods is essential for advancing treatment planning systems, particularly in medical imaging tasks like liver segmentation. Most quantum segmentation algorithms focus on translating classical methods into quantum formalisms. **Table 2** compares the performance of three quantum algorithms alongside AI-based methods, using Dice scores as the evaluation metric.

**Table 2:** Summary and comparison of quantum algorithms.

Model	Dice
QCUTS	0.2533 ± 0.25290
QIS-Net	0.3144± 0.1428
Grover AI	0.9309± 0.0400
AI	0.9307± 0.0383

QIS-Net was adapted from a region-growing strategy that relies on pixel intensity and local neighborhood information to guide expansion. The region-growing method was modeled as a logic neural network that was converted to a quantum-inspired Fuzzy logic algorithm. The main challenge with this method is the requirement of choosing, through trial and error, the activation function for the qutrit and the qutrit translation function, $F^{\lambda_{\omega L}}\in [0,\frac{\pi }{2}$ ]. We tried optimizing the algorithm over a range of values for these two parameters to improve the performance, but the Dice score remained low. Region-growing methods, such as the one used in QIS-Net, struggle with low-contrast boundaries, noise, and the need for carefully tuned parameters—limitations that remained even after incorporating quantum-inspired elements. The quantum adaptation did not resolve these fundamental challenges, resulting in poor segmentation performance. The reliance on trial-and-error optimization further reduces its practicality for treatment planning.

The quantum annealer algorithm implements graph-based image segmentation, which can also be solved classically using simulated annealing with equivalent results. The primary advantage of the quantum annealer lies in its potential for acceleration rather than improved accuracy. In our study, converting the classical graph approach into a quantum annealing framework did not overcome these limitations. The method remains useful for high-contrast regions. Enhancements, such as incorporating a pixel region of interest (ROI) for the liver as the foreground, could improve its performance. This would require either manual ROI selection or AI-based auto-contouring. While QCUTS was not designed for abdominal medical imaging, its implementation serves as a valuable example for medical physicists exploring quantum annealer hardware. QCUTS achieved a Dice score of 0.2533 ± 0.2529, reflecting its limited applicability in this context.

Grover’s algorithm was implemented on a qubit-based quantum computer. It used predictions from an AI auto-contouring liver model to search for the quantum-liver state (marked state). As a metaheuristic algorithm, Grover’s method offers an approximate solution akin to thresholding post-processing. Although it slightly improved the Dice score (0.9309± 0.0400) compared to AI auto-contouring alone (0.9307± 0.0383), the improvement was within the standard deviation, rendering it statistically insignificant. Repeated experiments using different seeds for AI auto-contouring confirmed this finding.

Both QIS-Net and QCuts demonstrated performance challenges that go beyond simple contrast limitations. QIS-Net, which relies on fuzzy logic applied to local pixel neighborhoods, underperformed in cases with heterogeneous textures and ambiguous anatomical boundaries. These characteristics reduced the effectiveness of its qutrit-based activation and translation functions, even after parameter optimization. In contrast, QCuts is designed for high-contrast or binary (black-and-white) images and relies heavily on grayscale intensity differences to define graph edges. As a result, it struggled with the continuous grayscale profiles common in abdominal MR images, where liver boundaries are often subtly defined. To illustrate these algorithm-specific limitations, we selected a representative patient case (1-artierial,2-pre-contrast,3-venous) where both QIS-Net and QCuts yielded low Dice scores and integrated this example across **Figures 2** (QIS_NET), **Figure 5** (AI baseline), **Figure 8** (Grover AI), and **Figure 11** (QCuts). This allows for a direct visual comparison across all evaluated methods and highlights areas where classical AI and quantum-classical hybrids currently outperform purely quantum-inspired or annealing-based methods. These qualitative insights help contextualize the observed performance gaps and suggest clear targets for future refinement.

In terms of potential hardware implementation, the quantum resource demands for Grover’s segmentation and QCuts differ considerably. For Grover’s algorithm, a 64×64 image requires at least 12 qubits using NAQSS encoding to map pixel data into quantum state space. In contrast, a D-Wave implementation of QCuts—assuming the image is downsampled into 256 superpixels—would require approximately 256 logical qubits to represent the graph-based segmentation problem. Due to D-Wave’s sparse physical qubit connectivity, this could translate to 384–2560 physical qubits after accounting for embedding overhead. These estimates underscore the practical differences in hardware scalability and implementation pathways between quantum circuit-based and quantum annealing approaches.

Overall, while quantum segmentation methods like QCUTS, QIS-Net, and Grover AI demonstrate innovative approaches, they face significant challenges in accuracy, efficiency, and adaptability. Current results suggest that further refinement and integration with classical AI methods are needed to realize their full potential in medical imaging applications.

**Figure 13:**
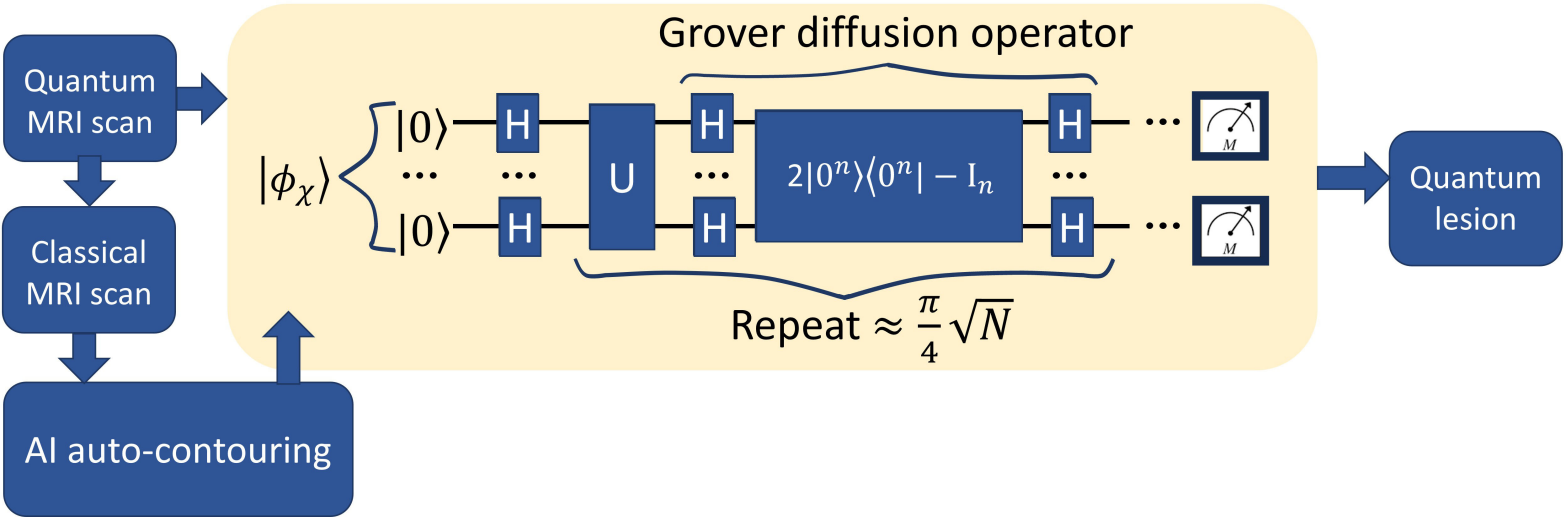
Possible Quantum image segmentation.

Of the three algorithms that we implemented and evaluated on MR images, we found that the inclusion of AI into the Grover’s quantum algorithm yielded the best results. This is mainly due to the fact that casting classical algorithms in a quantum space doesn’t necessarily mean that it improves the accuracy of the classical method. While Grover’s algorithm is not necessary for classical image segmentation, it may allow for a means to perform quantum image segmentation on actual quantum medical images. See **Figure 13**. The generated quantum medical image could be cloned with non-perfect fidelity into a classical image for AI to mark the states, then converted back into quantum information (as quantum marked states). Grover’s algorithm would search for the quantum states within the quantum generated image and perform auto-contouring, while maintaining the quantum information. This would perform auto-contouring in a quantum space rather than in a classical space.

## 9 Practical clinical implications

Quantum computing offers theoretical capabilities that could address some of the limitations of traditional AI-based segmentation methods including, static model parameters, deterministic outputs, and challenges to anatomical uncertainties with outliers within datasets. Quantum systems inherently encode probabilistic information, which may allow for more flexible handling of uncertainty in image segmentation, particularly in anatomically complex or ambiguous regions. Such capabilities, if realized, could be particularly valuable in adaptive radiotherapy workflows where frequent re-contouring based on daily imaging is required. In the context of treatment planning for radiation therapy, the ability to rapidly and accurately auto-contour organs at risk and target structures is critical for improving efficiency and maintaining precision.

Our current evaluation of quantum segmentation methods, however, demonstrates that they are not yet clinically competitive with classical AI approaches. This is likely because most quantum segmentation algorithms today are adaptations of classical techniques rather than fundamentally quantum-native designs, limiting their ability to offer distinct advantages. Moreover, although pre-processing steps such as bias field correction and intensity normalization were applied to harmonize images across scanners, scanner-specific variability was not analyzed separately due to limited sample sizes within each subgroup. This remains a potential source of variation in segmentation performance and will be addressed in future studies.

In terms of computational practicality, we also observed significant overhead in simulating quantum algorithms on classical machines. For example, Grover’s algorithm was the most computationally intensive due to the large memory and matrix operations needed for simulating amplitude amplification. Both QCuts and QISNET also matrix operations intensive. In contrast, the classical AI baseline ran efficiently on a high-performance GPU. These results suggest that, while quantum algorithms may one day offer novel capabilities, their current simulation costs are substantially higher and not yet viable for routine clinical deployment.

Finally, even if technical challenges are overcome, integrating quantum algorithms into clinical treatment planning systems will require addressing additional barriers, including software validation, regulatory approval, and ensuring interpretability for clinical end users. Even if technical hurdles are overcome, integrating quantum algorithms into clinical treatment planning systems would require addressing significant barriers related to software validation, regulatory approval, and clinician interpretability.

One promising future direction lies in leveraging the inherent probabilistic structure of quantum systems to generate voxel-wise uncertainty maps during segmentation. Such maps could highlight ambiguous regions where manual review is warranted—an area where classical deep learning models often fall short. This capability could ultimately improve trust and safety in auto-contouring pipelines, especially in anatomically complex or borderline cases.

## 10 Concluding remarks

A significant amount of progress remains to be made before practical use of a quantum computer can be implemented in medical imaging. Current quantum segmentation algorithms, including those evaluated in this study, do not demonstrate a clear advantage over established classical methods in terms of segmentation accuracy or clinical applicability. While improvements in quantum hardware—such as increasing qubit counts, enhancing error correction, and developing quantum data storage and communication—are critical for future development, it is equally important to recognize that advances cannot rely solely on expectations of faster computation. Future research must explore how uniquely quantum properties, such as probabilistic encoding and entanglement, can be leveraged to address clinical challenges that classical systems struggle with, including handling uncertainty in image segmentation and supporting adaptive treatment workflows. Quantum computing offers the ability to present medical image pixel values as a probability. Currently, distinguishing different tissue types, such as muscle and fat, on CT scans is difficult. Quantum representation of the pixel values would allow for algorithms to leverage Hounsfield units as a probability for image segmentation. Evaluation of the performance of quantum algorithms in processing medical images would help Medical Physicists understand capabilities of quantum computing in medical radiology and diagnostic imaging.

## Data Availability

The data analyzed in this study is subject to the following licenses/restrictions: The Dataset is an internal MD Anderson dataset. Requests to access these datasets should be directed to DTFuentes@mdanderson.org.
